# Does the Temperature of the *prise de mousse* Affect the Effervescence and the Foam of Sparkling Wines?

**DOI:** 10.3390/molecules26154434

**Published:** 2021-07-22

**Authors:** Clara Cilindre, Céline Henrion, Laure Coquard, Barbara Poty, Jacques-Emmanuel Barbier, Bertrand Robillard, Gérard Liger-Belair

**Affiliations:** 1 Université de Reims Champagne-Ardenne, CNRS, GSMA UMR 7331, 51097 Reims, France; laure.coquard@gmail.com (L.C.); barbara.poty@univ-reims.fr (B.P.); gerard.liger-belair@univ-reims.fr (G.L.-B.); 2 Institut Œnologique de Champagne (IOC), ZI de Mardeuil, Epernay, 51530 Mardeuil, France; chenrion@iocwine.com (C.H.); jebarbier@iocwine.com (J.-E.B.); brobillard@iocwine.com (B.R.)

**Keywords:** sparkling wine, Champagne, Crémant, *prise de mousse*, CO_2_, effervescence, bubble, foam, glass, tasting

## Abstract

The persistence of effervescence and foam collar during a Champagne or sparkling wine tasting constitute one, among others, specific consumer preference for these products. Many different factors related to the product or to the tasting conditions might influence their behavior in the glass. However, the underlying factor behind the fizziness of these wines involves a second in-bottle alcoholic fermentation, also well known as the *prise de mousse*. The aim of this study was to assess whether a low temperature (13 °C) or a high temperature (20 °C) during the in-bottle fermentation might have an impact on the effervescence and the foaming properties (i.e., collar height and bubble size) of three French sparkling wines (a Crémant de Loire and two Champagne wines), under standard tasting conditions. Our results showed that sparkling wines elaborated at 13 °C and served in standard tasting conditions (i.e., 100 mL, 18 °C) had better ability to keep the dissolved CO_2_ (between 0.09 and 0.30 g/L) in the liquid phase than those elaborated at 20 °C (with *P* < 0.05). Most interestingly, we also observed, for the Crémant de Loire and for one Champagne wine, that the lower the temperature of the *prise de mousse*, the smaller (with *P* < 0.05) the bubbles in the foam collar throughout the wine tasting.

## 1. Introduction

**Figure 1:**
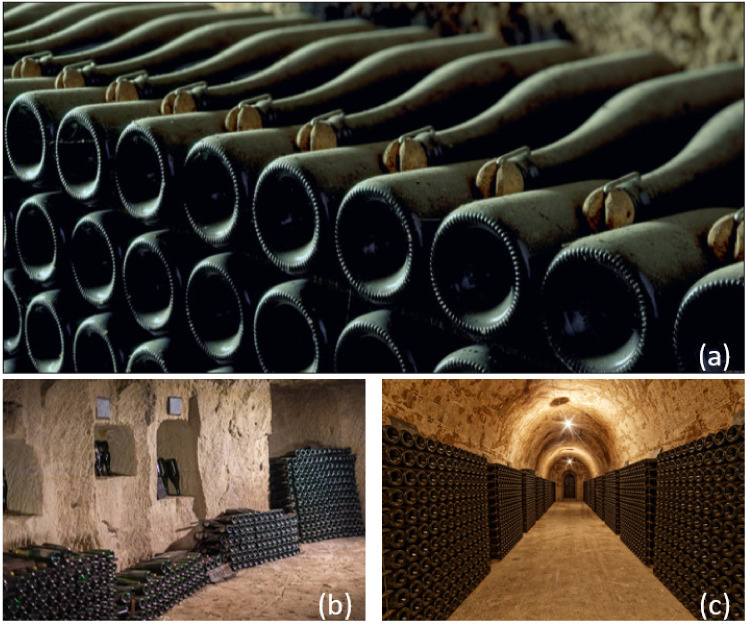
(**a**) Bottles of Champagne hermetically sealed with a natural cork and stacked horizontally in the course of their *prise de mousse*, followed by a long period of ageing on lees (Collection CIVC). Crémant de Loire and Champagne undergoing their *prise de mousse* in cellars (**b**) from the Loire area (Gratien et Meyer) and (**c**) from the Champagne area (Emmanuel Goulet).

Champagne and sparkling wines from around the world, elaborated through the traditional method, are supersaturated with CO_2_ which is forced to dissolve progressively in the liquid phase during a second in-bottle alcoholic fermentation, the so-called *prise de mousse* (literally “capturing the sparkle”). This step is followed by a minimum aging period of 9 or 15 months in contact with the lees, according to the sparkling wine produced, as for example Crémant de Loire and Champagne, respectively (Figure 1). Actually, a standard 75 cL Champagne or sparkling wine bottle typically holds about 8–9 g of dissolved CO_2_ (i.e., ≈10–12 g/L^−1^). This content of dissolved CO_2_ in the liquid phase is responsible for bubble formation once the bottle is uncorked and the wine poured into a glass [1]. Dissolved CO_2_ is a parameter of high importance since it directly impacts the following sensory properties: the frequency of bubble formation, the growth rate of rising bubble, the very characteristic tingling sensation in mouth, and the overall olfactory perception of Champagne and sparkling wines [1]. Moreover, Champagne and sparkling wines also contain many compounds originating from the grape and yeast that might influence the stability of bubbles and the height of the foam collar in the glass [2]. Among them, wine proteins are considered important components for the foam stability of sparkling wines [3,4].

During the *prise de mousse* and the autolysis that occurs during the ageing of the wine, yeasts are involved in many biochemical reactions, and changes in the chemical composition of the wine, through the release of compounds from the yeast and also through the formation and transformation of molecules and macromolecules that contribute to the organoleptic quality of the wine [5,6]. Numerous parameters that contribute to the completion of this secondary fermentation have been studied, as recently reviewed by Kemp et al. (2015) [6]. Indeed, specific commercial dry yeast strains are usually well adapted to all and every parameter related to the composition of the changing fermented wine [7,8]. As for example, their tolerance towards several stress factors, such as high levels of ethanol and SO_2_, CO_2_ overpressure, nitrogen deficiency, low pH and low temperature, is essential in order to drive the secondary fermentation to successful completion [6,7,9,10,11]. Recently, the impact of CO_2_ overpressure has been revealed, through proteomics studies, on the metabolism of yeast and on the proteins involved in the response to stresses during the second alcoholic fermentation [12,13]. Such approaches could be used as a tool to select the appropriate strains of yeast to conduct the *prise de mousse* as a function of the organoleptic characteristics of the final product. Martínez-García et al. (2017) [14] have also demonstrated the effect of CO_2_ overpressure on the typical aromas of sparkling wines released during the *prise de mousse*. Another important parameter that might influence the kinetics of the *prise de mousse* and the metabolism of yeast, is the temperature, but it has not been dealt with in depth.

In France, from a regulatory point of view, there is no obligation to conduct the *prise de mousse* at a specific temperature (nor for the hygrometry). The National Institute of origin and quality (INAO) supervises the specifications of French sparkling wines produced with an official sign of quality such as Champagne and cCémant de Loire. Only historical references mention the advantages of using cellars dug in the tuffeau of the Loire (Figure 1b) or in the chalk (chalk pits) in the Champagne area (Figure 1c), to develop the production of Crémant de Loire and Champagne, and above all to conduct a slow *prise de mousse* at a low temperature [15,16]. Until the first half of the 20th century, the second fermentation conducted in cellars, between 10 and 15 °C, also made it possible to limit the breakage of bottles which were less pressure-resistant and the *prise de mousse* process being less controlled than nowadays [17,18].

Nevertheless, in the Champagne area and for decades, it has often been said about the *prise de mousse* that: “if it is too rapid and it produces large, flabby bubbles that soon go flat. But taken slowly, at a cool even temperature, it leaves the wine with fine, delicate bubbles that seemingly last forever”, as mentioned on the official website of the Union of the Champagne Houses [19]. To the best of our knowledge, although the second in-bottle fermentation is traditionally carried out in cellars where the temperature is close to 13 °C and constant all year along, no studies have examined the relation between this parameter and the persistence and the finesse of the effervescence, or of the foam collar of the sparkling wines elaborated through the traditional method. Higher temperatures might likely influence the release of compounds by yeasts, as well as the chemical and biochemical reactions undergone by the wine. The first report on the influence of temperature (12 or 16 °C) during the second fermentation and aging of Spanish sparkling wines was conducted in 2015 by Esteruelas et al. [20]. Interestingly, better foaming properties were observed for the Cavas elaborated at a low temperature (i.e., 12 °C), which might be explained by their higher content in low molecular weight proteins (below 40 kDa) and oligosaccharides (below 7.5 kDa). However, the foaming properties were studied through a gas sparging method on a degassed sparkling wine, and thus far from real tasting conditions.

As such, the aim of our study was to unveil the impact of two distinct temperatures for the *prise de mousse* (namely, 13 and 20 °C) on several characteristics of sparkling wines, in real tasting conditions: (i) the progressive loss of dissolved CO_2_ as well as (ii) the collar height and (iii) the average bubble size in the collar. Three batches of base wines were used for these experiments: one Crémant de Loire and two Champagnes. A campaign of analysis was carried out on the wines that were disgorged two weeks after the end of the secondary fermentation.

## 2. Results

### 2.1. Initial Concentrations of Dissolved CO_2_

The first comparison on the influence of the temperature of the *prise de mousse* was made on a typical characteristic of Champagne or sparkling wines: their dissolved CO_2_ concentration (responsible for bubble formation and foam). As first observed by Liger-Belair et al. [21], losses of dissolved CO_2_ are indeed experienced by a Champagne wine during the pouring step, because turbulence and eddies vigorously agitate the liquid phase as Champagne progressively invades the glass. Immediately after pouring Champagne into a glass, the dissolved CO_2_ concentration falls to a level in a range between about 6 and 9 g.L^−1^, depending on several parameters, such as the Champagne temperature [22], bottle type [23], or glass shape [24]. Table 1 displays the average concentration of dissolved CO_2_ initially found in the bottle before pouring ($c_{bottle}$) and the one measured just after pouring each sample into a flute ($c_{0_{flute}}$).

Before pouring, samples A13 and A20 had a similar $c_{bottle}$ whereas for samples B and C, the concentration of dissolved CO_2_ found in the bottle was higher in the Champagne wines elaborated at 20 °C for their *prise de mousse* than those elaborated at a lower temperature. Then, it was crucial to measure the initial concentration of dissolved CO_2_ in the flute, immediately after pouring each sample of wine, ($c_{0_{flute}}$). Indeed, it is preferable that each pair of compared wines hold the same concentration of dissolved CO_2_ in the flute, in order to provide a rigorous comparison of the loss of dissolved CO_2_ and of the foaming properties of these wines. We can observe from Table 1 that the initial dissolved CO_2_ concentrations found in the flute ($c_{0_{flute}}$) were statistically similar for each batch of sparkling wine. We can thus conclude that the temperature of the *prise de mousse* had no impact on this parameter, whatever the batch of sparkling wine.

### 2.2. Influence of the Temperature of the prise de mousse on the Losses of Dissolved CO_2_ versus Time

**Figure 2:**
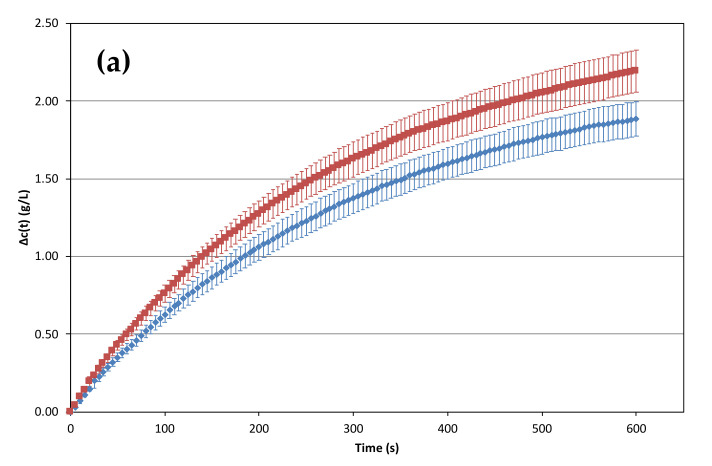

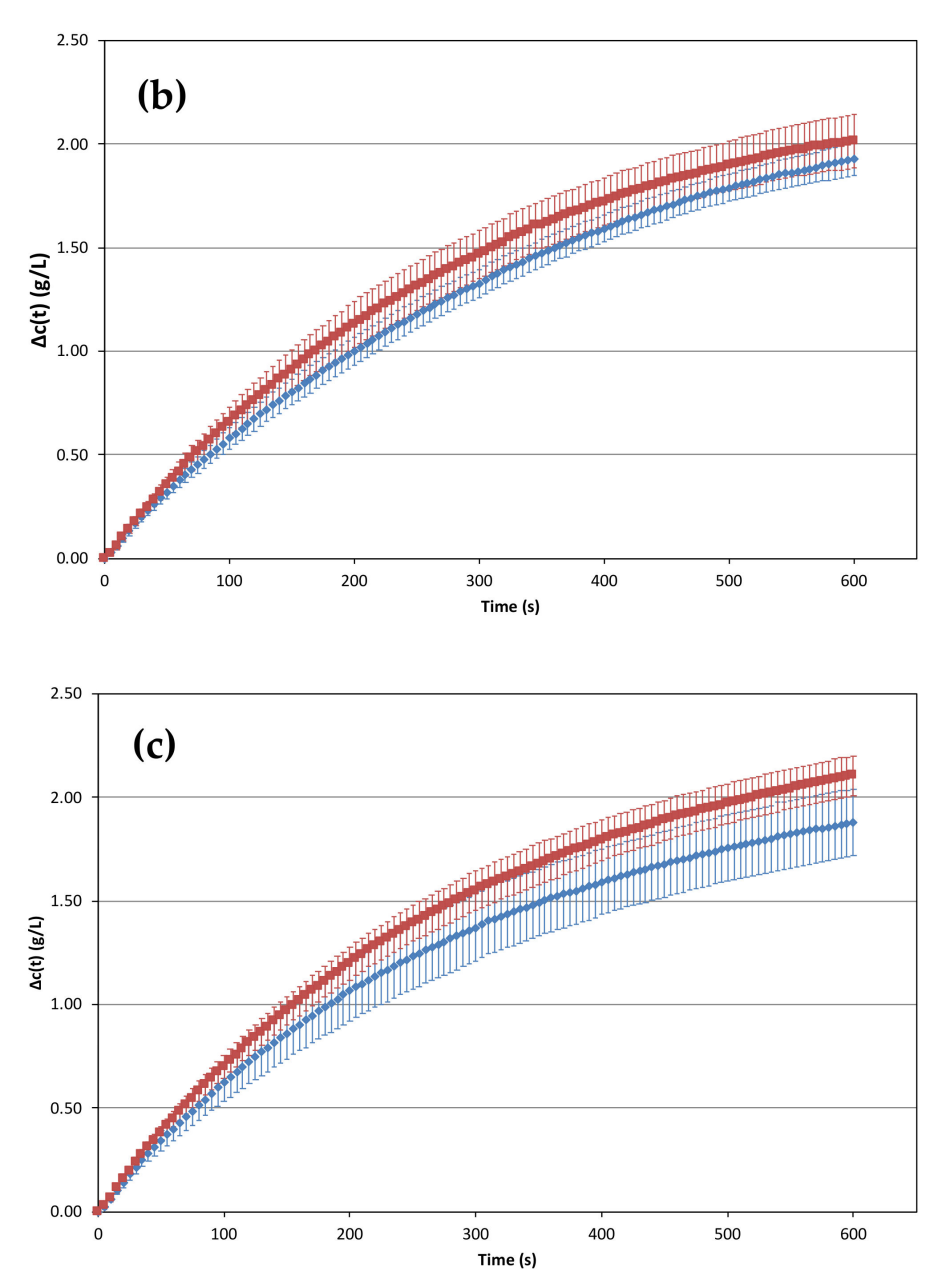
Progressive losses of dissolved CO_2_ concentration (g.L^−1^) during the ten minutes following pouring of each pair of sparkling wine into a flute; (**a**) sparkling wines A13 (blue, n = 12) and A20 (red, n = 10); (**b**) sparkling wines B13 (blue, n = 10) and B20 (red, n = 9); (**c**) sparkling wines C13 (blue, n = 11) and C20 (red, n = 10). Each point of each curve corresponds to the mean of values recorded from at least nine successive pourings. Error bars corresponds to standard deviations of the values obtained by the successive data recordings.

Once each sample of sparkling wine was poured into the flute, the progressive loss of dissolved CO_2_ concentration with time, denoted $\Delta c\left(t\right)\;$and expressed in g.L^−1^, was easily accessed by retrieving the following relationship established from the cumulative mass-time series ($m\left(t\right)$) corresponding to each sample of wine:

(1) $$\Delta c\left(t\right)=c_{flute}\left(t\right)-c_{0_{flute}}=-\frac{m\left(t\right)}{V_{flute}}$$

with $V_{flute}$ corresponding to the volume of sparkling poured into the flute in L (i.e., 0.1 L in these experiments). Losses of dissolved CO_2_ concentration experienced by each pair of sparkling wines (A13/A20; B13/B20; C13/C20) during the ten minutes following the pouring process, were calculated from the above equation and are presented in Figure 2.

It is worth noting that, for a given sparkling wine sample, the concentration of dissolved CO_2_ found within a flute progressively decreases in the course of the 10 min following pouring. As displayed in Figure 2a, for sparkling wine A, a significant difference appears between the two progressive loss–time curves of dissolved CO_2_ concentration. At 600 s, the cumulative loss of dissolved CO_2_ for sparkling A20 and A13, was 2.19 g/L and 1.89 g/L, respectively. Thus, a lower temperature of the *prise de mousse*, for this wine, allowed a significant reduction (*P* < 0.05) in the loss of dissolved CO_2_ during the ten minutes of this experiment. Indeed, sparkling wines B20 and C20 also suffered larger losses of dissolved CO_2_ than the corresponding wines elaborated at 13 °C for the *prise de mousse*, but differences were only significantly different (at *P* < 0.05) between 50 s and 600 s after the beginning of monitoring. Depending on the wine sample, between 0.09 and 0.30 g/L of dissolved CO_2_ can be kept in the liquid during the ten min of tasting, if the sparkling wine is elaborated at a lower temperature during the *prise de mousse*. As far as we know, this is the first time that the impact of the temperature of the *prise de mousse* was shown to reduce the loss of dissolved CO_2_ under standard tasting conditions.

### 2.3. Influence of the Temperature of the prise de mousse on CO_2_ Volume Fluxes

**Figure 3:**
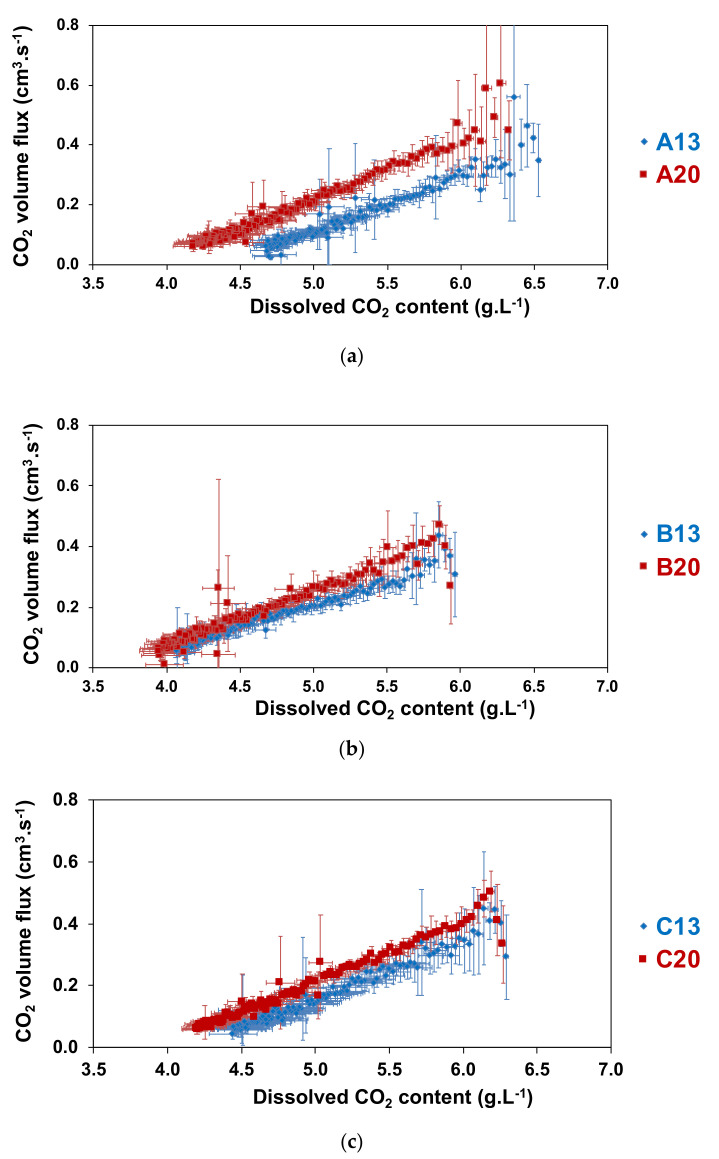
CO_2_ volume flux (in cm^3^.s^−1^) desorbing from a 100 mL flute filled with wine samples (**a**) A13 and A20, (**b**) B13 and B20, and (**c**) C13 and C20, as function of its dissolved-CO_2_ concentration (in g.L^−1^). Each data of each time series is the arithmetic average of at least nine successive values recorded from at least nine successive pourings.

Once poured into the flute, the driving force behind the desorption of dissolved CO_2_ from Champagne or sparkling wines being its bulk concentration of dissolved CO_2_, it seemed relevant to propose a correlation between the CO_2_ volume flux outgassing from the flute (see Section 4.3) and the continuously decreasing bulk concentration of dissolved CO_2_ (Figure 3) [25].

It is clear from Figure 3 that the higher the concentration of dissolved CO_2_ in a sparkling wine, the higher the CO_2_ volume flux escaping from the flute. Most interestingly, for a given dissolved CO_2_ concentration, the lower the temperature of the *prise de mousse*, the lower the CO_2_ volume fluxes found to be released from the flute. Nevertheless, this result was exclusively significant for more than half of the data for sparkling wines A, more than a third for sparkling wines C, and a few points for sparkling wines B.

Therefore, the lower the temperature of the *prise de mousse* of the wine, the lower the progressive loss of dissolved CO_2_ concentration with time. The observed increase in the CO_2_ volume fluxes for samples A20, B20 and C20 could be interpreted as being a result of bubbles with a higher diameter. The concentration of dissolved CO_2_ influences the effervescence and thus the CO_2_-degassing process of Champagne and sparkling wines. It is also correlated to the bubble size at the air–liquid surface that forms the foam collar [26]. 

To determine whether the temperature of the *prise de mousse* had an effect on bubble size, the evolution of the foaming properties (i.e., foam collar and bubble size) was studied under real tasting and standardized conditions, as described in the following paragraph.

### 2.4. Foaming Properties in Real Tasting Conditions

Various methods exist to study the foaming properties of Champagne and sparkling wines [27]. The widely used gas-sparging method, namely the Mosalux, has been previously employed to determine the influence of temperature during the second fermentation and aging of Spanish sparkling wines (AOC Cava) on various foam parameters (maximum foam height and stable foam height) [20]. They showed that a lower temperature during the *prise de mousse* (i.e., 12 °C) led to better foaming properties. In order to analyze the foaming properties of our samples in real tasting conditions, it was decided that the optimal procedure was to take pictures of the foam collar every 30 s during the ten minutes following the end of pouring.

#### 2.4.1. Foam Collar

**Figure 4:**
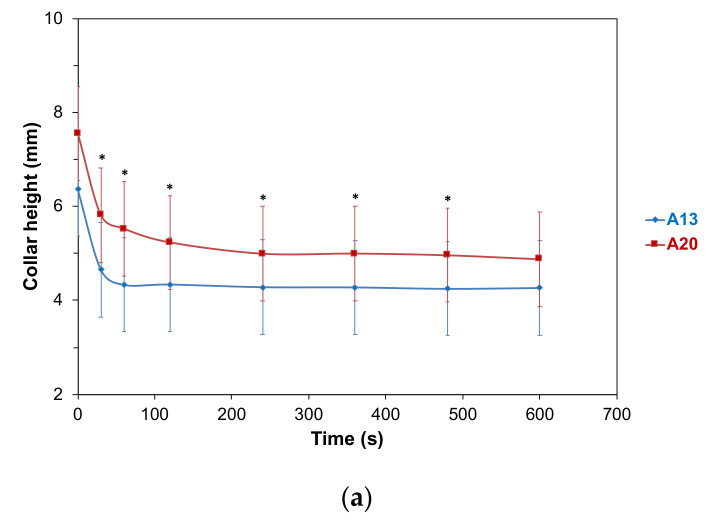

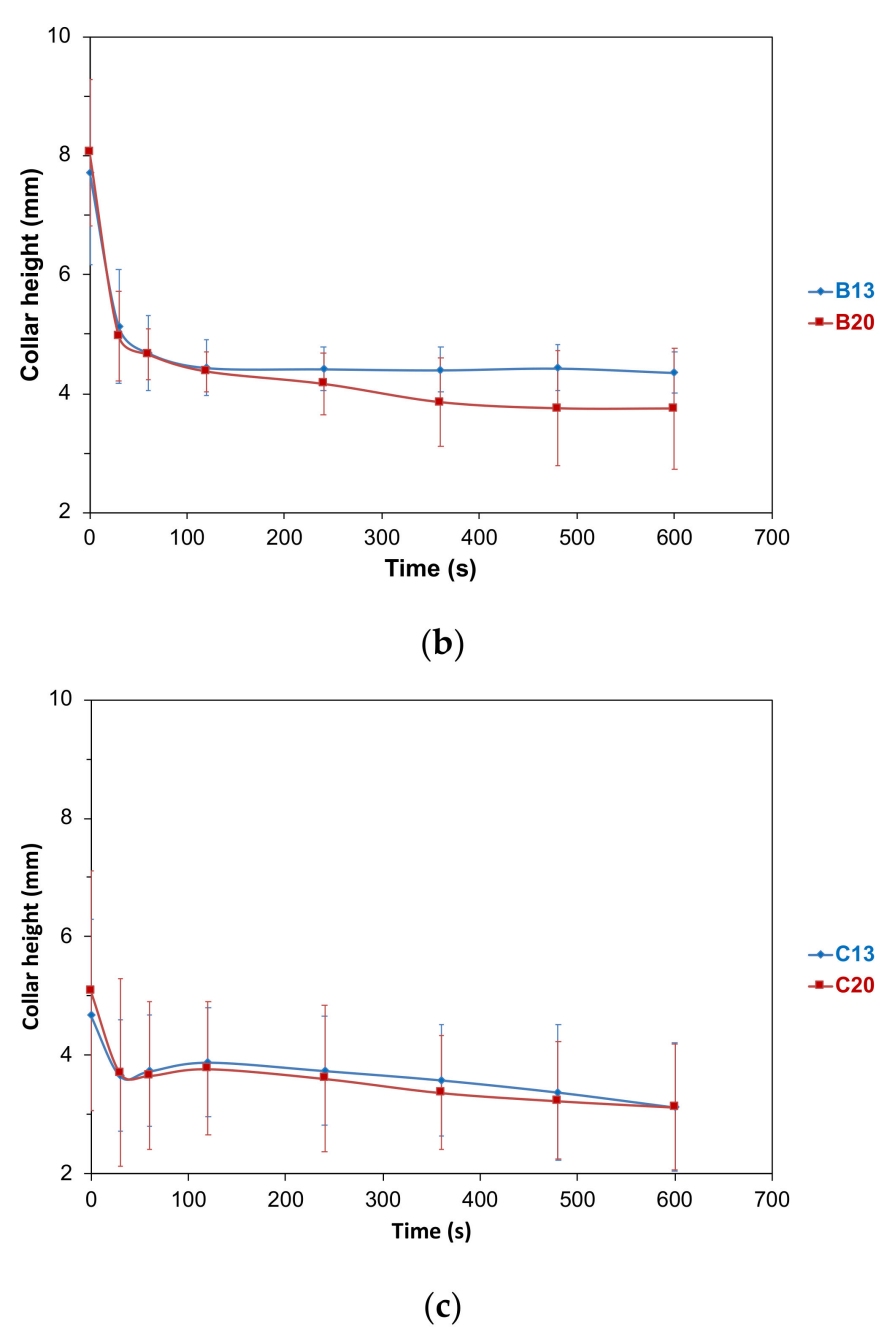
Evolution of the foam collar under standardized tasting conditions, during ten minutes after the end of pouring of (**a**) wine samples A, (**b**) wine samples B, and (**c**) wine samples C. Heights of foam that are significantly different, are denoted with an asterisk (with *P* < 0.05).

The so-called effervescence process of Champagne and sparkling wines leads to the formation of a foam ring at the periphery of the glass, also known as the collar or foam collar. The evolution of the foam collar of each sparkling wine was monitored during the first ten minutes following the end of pouring (Figure 4).

At 0 s, the height of the foam was often much higher, as it is most related to the amount of foam formed during the pouring of the sparkling wine into the glass. Depending on the sample, this foam might disappear more or less quickly. Furthermore, the amount of foam formed during the pouring process is dependent on the concentration of dissolved CO_2_ and also on the presence of molecules that are able to promote foam. In our experiments, all the laser-etched glasses provided a standardized effervescence, only restricted to the artificial nucleation sites.

The evolution of the foam collar was similar between samples B13 and B20 (Figure 4b) and between samples C13 and C20 (Figure 4c) for the ten minutes following the end of pouring. As seen in Figure 4a, the collar of sparkling wine A elaborated at 20 °C (A20) was significantly thicker than the collar of the same wine elaborated at 13 °C (A13), between 30 s and 480 s after the end of pouring. Thus, our study performed under real tasting conditions did not confirm previous results from Esteruelas et al. (2015) [20] obtained with the Mosalux method.

#### 2.4.2. Bubble Size

**Figure 5:**
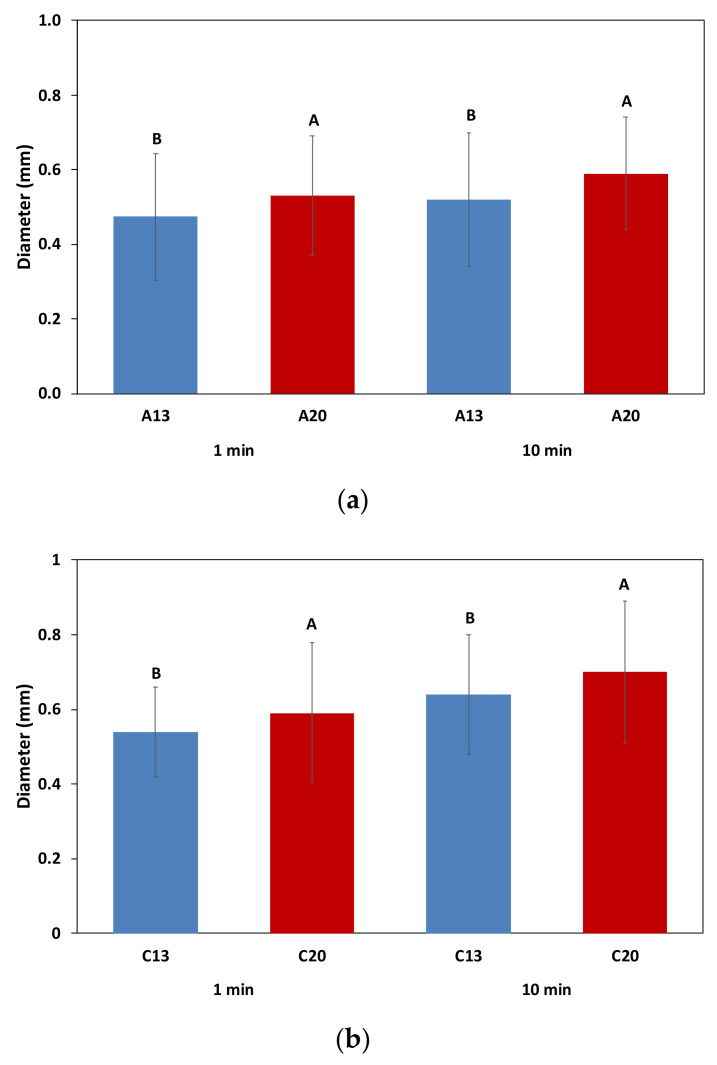
Average diameter of bubbles in the foam collar from the six wine samples, measured at 1 and 10 min after the end of pouring. (**a**) Wine samples A and (**b**) wine samples C.

The diameter of the bubbles in the foam collar was measured at 1 min after the end of pouring and at 10 min (Figure 5). One minute was chosen as it corresponds approximately to the end of the collapse of the foam after the pouring process, and to the beginning of a rather stable plateau related to the foam stability. Then, as the foam collar remained stable until the end of the experiment (as seen in Figure 4), it was interesting to compare the bubble sizes at 10 min, in order to examine if the temperature of the *prise de mousse* might also have an impact on it.

At 1 min after the end of pouring (Figure 5a,b), the average diameter of the bubbles in the foam collar was significantly higher for the wines A20 and C20. Furthermore, as seen in Figure 5, the bubbles retained in the foam collar, at 10 min after the end of pouring, were also significantly smaller for the wines A13 and C13, elaborated at a lower temperature during the *prise de mousse*. No significant difference was observed for the bubbles of the wines B13 and B20 (data not shown). We also observed that, rather counterintuitively, the size of the bubbles in the foam collar increased during the ten min after pouring. Indeed, Liger-Belair showed in 2005 that the average size of the bubbles rising to the liquid surface progressively decreases as time proceeds in a glass of Champagne or sparkling wine, due to the progressive loss of dissolved CO_2_ from the liquid (through the effervescence and through the diffusion of CO_2_ across the air/wine interface) [26]. Interestingly, in 2015, White and Heyman [28], also showed a decrease in the visual effervescence attributes (i.e., size and concentration of bubbles in the liquid) over time. In our case, we have followed the size of the bubbles that form the foam collar. We can thus suppose that the chemistry of these sparkling wines leads to a better stability of the bubbles, and thus leads to a higher size at 10 min after the end of pouring, whatever the temperature of the *prise de mousse*. This might be the result of a good balance between the bubble coalescence, bubble disproportionation and foam drainage that would occur at a lower rate [27]. Moreover, the presence of tensioactive macromolecules in the liquid film (such as proteins and polysaccharides) would lead to an expansion of the CO_2_ inside the bubbles, leading to larger bubbles with a better stability.

**Figure 6:**
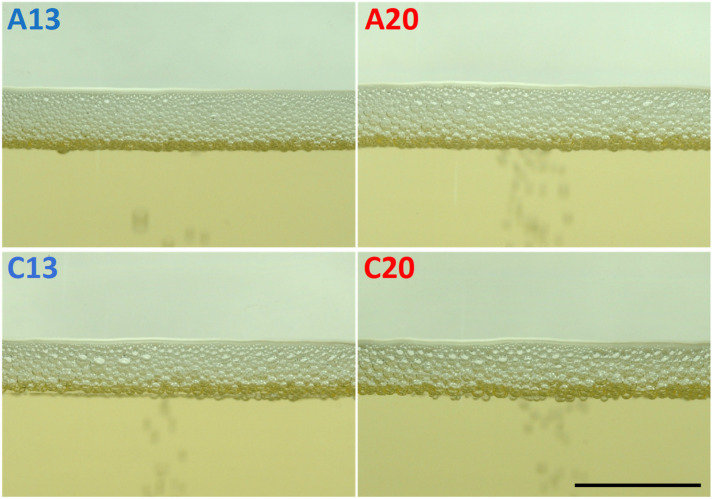
Closeup of the foam collar at 10 min after the end of pouring, from wine samples A and C (bar = 1 cm).

Finally, the photographs displayed in Figure 6 compare the foam collar from the batches of sparkling wines A and C, at 10 min after pouring. It is clear that the bubble’s size distribution is different between the wines elaborated at 13 °C for the *prise de mousse* and those elaborated at 20 °C. Thus, A13 and C13 showed significantly smaller bubbles than the wines A20 and C20, respectively. These differences observed between the wines elaborated at 13 °C and those elaborated at 20 °C might be explained by the differences in the chemical composition that occurred during the period of the in-bottle fermentation [29,30]. Indeed, we have noted that the quantity of total yeast cells at the end of the *prise de mousse* was much lower at 13 °C than at 20 °C, for sparkling wines A and C (see Figure S1). Nevertheless, viable yeast cells were only present in the sparkling wines elaborated at 13 °C. Thus, the quantity and, also the nature of the compounds released by these yeast populations might vary according to the final yeast population (and to the proportion of viable and dead yeast cells), but also thanks to the effect of the temperature of the *prise de mousse* that should accelerate the autolytic process [5,9,10].

## 3. Conclusions

The “*prise de mousse*” is a key step of the traditional method, which has been thoroughly studied since the end of the 17th century, from the first elaboration of sparkling wines in Champagne and worldwide. Many renowned scientists and men have contributed to acquire better knowledge and control of this second alcoholic fermentation, enabling its success in order to obtain high quality sparkling wines [17].

As far as we know, this is the first set of analyses that investigated the impact of the temperature of the *prise de mousse* on the effervescence and the foaming properties under standard tasting conditions. Our results showed that the sparkling wines elaborated at 13 °C and served in standardized tasting conditions had a better ability to keep the dissolved CO_2_ in the liquid phase than those elaborated at 20 °C (with *P* < 0.05). Most interestingly, we also observed that two batches of sparkling wines (one Crémant de Loire and one Champagne wine) which underwent their *prise de mousse* at 13 °C presented slightly smaller bubbles in the foam collar (with *P* < 0.05). Further experiments are under investigation to confirm these results on Champagne wines and sparkling wines aged during a longer period (i.e., 15 months). A detailed knowledge of the chemical and biochemical differences between the sparkling wines elaborated at 13 °C and 20 °C may help to better understand the different behaviors observed in this study.

## 4. Materials and Methods

### 4.1. Sparkling Wines Samples

Three batches of sparkling wines, namely A, B and C, were elaborated from three distinct base wines. Sample A correspond to a base wine from Crémant de Loire and samples B and C are Champagne base wines. All base wines were elaborated under standard winemaking practices. The same conditions (yeast IOC 18-2007, IOC, France; sucrose; caps PE.DI, Ivrea, Italy) were applied for the 2nd in-bottle alcoholic fermentation to the three batches of base wines, except for the temperature. Indeed, each batch was divided into two groups: one elaborated at 13 °C, and defined as A13, B13 and C13; and another group elaborated at 20 °C (i.e., A20, B20 and C20). All wines were disgorged manually two weeks after the end of the 2nd alcoholic fermentation, and stored at their respective temperatures, 13 °C or 20 °C for the two weeks before the pouring experiment. Enological parameters were determined, through standard OIV analytical methods, on one bottle for each sparkling wine (see Figure S2).

### 4.2. Standardized Tasting Conditions

For the determination of the CO_2_ content in the flute and the measurement of the flux of CO_2_ desorbing from the flute poured with sparkling wines, 100 mL of each sample were poured into a standard flute (Lehmann glass, Reims, France). In order to provide a standardized effervescence for each sample, the flute was engraved at its bottom with twenty laser beam impacts. Prior to each experiment, the flute was thoroughly washed in a dilute acetic acid solution, rinsed using hot tap water and distilled water, and then oven-dried at 60 °C. This cleaning procedure forbids the formation of tartrate crystals on the glass wall as well as the adsorption of any dust particle acting as “natural” bubble nucleation sites. In this way, the CO_2_ bubble nucleation process is essentially restricted to the laser beam impacts, so that differences in CO_2_ release are attributed only to physicochemical differences between sparkling samples themselves. For 24 h prior to the pouring experiments, all sparkling wine samples were stored at 18 ± 1 °C. This temperature was chosen in order to avoid condensation on the glass wall, which would modify the mass loss of the flute and prevent a correct observation of the foam collar.

### 4.3. Measuring Concentrations of Dissolved CO_2_ in Sparkling Wines Samples

The determination of the dissolved CO_2_ concentration (in g.L*^−^*^1^) was achieved by the official method recommend by the International Office of Vine and Wine (OIV), based on the article by Caputi et al. (1970), using carbonic anhydrase (Sigma-Aldrich, USA) [31]. The dissolved CO_2_ content of each sparkling wine sample was measured in-bottle (*c_bottle_*) and, just after pouring the wine in the flute (*c_flute_*). Both measurements techniques were thoroughly described in Moriaux et al. (2021) [32].

### 4.4. Measuring the Flux of CO_2_ Desorbing from the Flute Poured with Sparkling Wines

A measure of 100 ± 3 mL of Champagne were carefully poured into a flute. Just after pouring, the glass was then manually placed on the weighing chamber base plate of a precision weighing balance (Sartorius, Secura 324 1S) with a total capacity of 320 g and a standard deviation of ±0.0001 g. The Sartorius balance was interfaced with a laptop PC recording data every 5 s from the start signal, activated just before the glass poured with sparkling wine was placed on the weighing chamber base plate. The total cumulative mass loss experienced by the glass poured with sparkling wine was recorded during the first 10 min following the pouring process. Actually, the mass loss of the flute poured with sparkling wine sample is the combination of both (i) CO_2_ progressively desorbing from the supersaturated liquid phase, and (ii) its evaporation. The mass loss attributed to sparkling wine evaporation only was accessible by recording the mass loss of a flute poured with a sample of 100 mL of sparkling wine first degassed under vacuum. 

Experiments were conducted in a thermo-regulated room with a constant temperature of 22 ± 1 °C. Between the successive pourings, bottles were hermetically closed and stored at 18 ± 1 °C. Due to likely variations in hygrometric conditions from day-to-day, sparkling wine evaporation was measured with a degassed sparkling wine sample, just before each series of total mass loss recordings were carried out. The cumulative mass loss versus time attributed only to CO_2_ molecules progressively desorbing from sparkling wine may therefore be readily available by subtracting the data series attributed to evaporation only from the total mass loss data series. Moreover, from a cumulative mass loss–time curve, the mass flux of CO_2_ desorbing from the sparkling wine surface (denoted $F_{CO_{2}}$) was thus experimentally deduced all along the degassing process in the flute, by dividing the mass loss Δm between two data recordings by the time interval Δt between two data recordings (i.e., $F_{CO_{2}}=\frac{\Delta m}{\Delta t}$). In champagne and sparkling wine tasting, it is nevertheless certainly more pertinent to deal with volume fluxes rather than with mass fluxes of CO_2_. By considering gaseous CO_2_ desorbing from sparkling wine as an ideal gas, the experimental total volume flux of CO_2_ (in cm^3^ s^−1^), denoted $F_{T}$, is therefore deduced as follows, all along the degassing process:

(2) $$F_{T}=10^{6}\left(\frac{RT}{MP}\right)\frac{\Delta m}{\Delta t}$$

with *R* being the ideal gas constant (equal to 8.31 J K^−1^ mol^−1^), *T* being the sparkling wine temperature (i.e., 291 K in the present case), *M* being the molar mass of CO_2_ (equal to 44 g mol^−1^), *P* being the ambient pressure (close to 10^5^ N m^−2^), the loss of mass between two successive data records Δm being expressed in g, and Δt being the time interval between two data recordings (i.e., 5 s in this case).

To enable a statistical treatment, at least three successive pourings from two distinct bottles and time series data recordings were done, for each sparkling wine (A13, A20, B13, B20, C13, C20). At each step of the time series (i.e., every 5 s), an arithmetic average of the six data provided by the six successive time series was calculated, to finally produce one single “average” data series (with standard deviations corresponding to the root-mean-square deviations of the values provided by the six successive data recordings).

### 4.5. Foaming Properties (Collar Height and Bubbles Size) in Tasting Conditions

**Figure 7:**
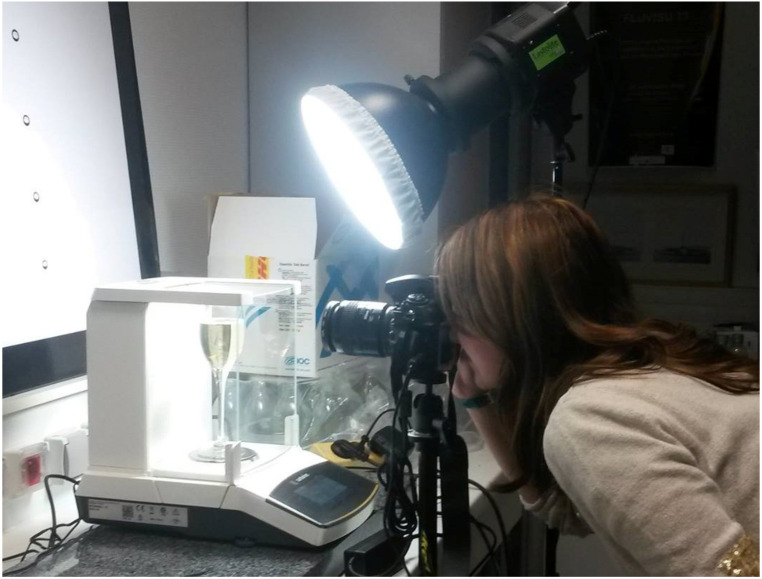
Experimental setup used to monitor simultaneously fluxes of gaseous CO_2_ and foaming properties, under standard tasting conditions.

Fluxes of gaseous CO_2_ released from a sparkling wine flute and foaming properties (collar height and bubble size) were simultaneously monitored under standard tasting conditions, throughout the 10 min following pouring (Figure 7).

Indeed, collar height and bubble size were measured through a collection of images captured by a digital camera every 30 s, during the 10 min following the pouring process. Collar height was precisely measured at 0, 30, 60, 120, 240, 360, 480 and 600 s, and bubble sizes of the foam collar were measured at 1 min and 10 min after the end of pouring. All measurements were performed using the ImageJ software (National Institute of Health, Bethesda, MD, USA, https://imagej.nih.gov/ij/, accessed on 21 July 2021).

### 4.6. Statistical Analysis

A one-way ANOVA with Student’s t-test using JMP Pro 12 software (SAS Institute) was carried out to determine whether average concentrations of dissolved CO_2_, fluxes of gaseous CO_2_, collar height and bubble size were considered as statistically different from sparkling wines elaborated at 13 °C to those elaborated at 20 °C. Differences at *P* < 0.05 were considered as significant.

## Figures and Tables

**Table 1:** Initial concentrations of dissolved CO_2_ found in the bottle ($c_{bottle}$) and in the flute ($c_{0_{flute}}$).

Concentration of Dissolved CO_2_ (g.L^−1^) ^1^	Temperature of the *prise de mousse* (°C)	Batch A	Batch B	Batch C
$c_{bottle}$	13	9.65 ± 0.07 ^a^	8.79 ± 0.08 ^a^	9.38 ± 0.08 ^b^
	20	9.60 ± 0.08 ^a^	8.92 ± 0.08 ^a^	9.52 ± 0.09 ^a^
$c_{0_{flute}}$	13	6.50 ± 0.23 ^a^	5.90 ± 0.16 ^a^	6.32 ± 0.16 ^a^
	20	6.37 ± 0.18 ^a^	5.96 ± 0.24 ^a^	6.30 ± 0.08 ^a^

^1^ Data represent mean value ± standard deviation (n = X). Values with different letters, within a column and for a concentration ($c_{bottle}$ or $c_{0_{flute}}$) are significantly different (*P* < 0.05).

## Data Availability

The datasets generated during and/or analysed during the current study are available from the corresponding author on reasonable request.

## Supplementary Materials

The following are available online, Figure S1: Yeast population at the end of the *prise de mousse*.

## Sample Availability

All samples of the compounds are available from the authors.

## References

[1] Liger-Belair G. (2017). Effervescence in Champagne and sparkling wines: From grape harvest to bubble rise. Eur. Phys. J. Spec. Top..

[2] Kemp B., Condé B., Jégou S., Howell K., Vasserot Y., Marchal R. (2019). Chemical compounds and mechanisms involved in the formation and stabilization of foam in sparkling wines. Crit. Rev. Food Sci. Nutr..

[3] Cilindre C., Fasoli E., D’Amato A., Liger-Belair G., Righetti P.G. (2014). It’s time to pop a cork on champagne’s proteome!. J. Proteomics.

[4] Condé B.C., Bouchard E., Culbert J.A., Wilkinson K.L., Fuentes S., Howell K.S. (2017). Soluble protein and amino acid content affects the foam quality of sparkling wine. J. Agric. Food Chem..

[5] Alexandre H., Guilloux-Benatier M. (2006). Yeast autolysis in sparkling wine—A review. Aust. J. Grape Wine Res..

[6] Kemp B., Alexandre H., Robillard B., Marchal R. (2015). Effect of production phase on bottle-fermented sparkling wine quality. J. Agric. Food Chem..

[7] Benucci I., Liburdi K., Cerreti M., Esti M. (2016). Characterization of active dry wine yeast during starter culture (*pied de cuve*) preparation for sparkling wine production. J. Food Sci..

[8] Laurent M., Valade M. (2007). La préparation du levain de tirage à partir de levures sèches actives. Le Vigneron Champenois.

[9] Valade M., Laurent M. (1999). La prise de mousse: Les phénomènes microbiologiques (1eère partie). Le Vigneron Champenois.

[10] Valade M., Laurent M. (1999). La prise de mousse: Les pheénomeènes microbiologiques (2eème partie). Le Vigneron Champenois.

[11] Penacho V., Valero E., Gonzalez R. (2012). Transcription profiling of sparkling wine second fermentation. Int. J. Food Microbiol..

[12] González-Jiménez M.d.C., García-Martínez T., Puig-Pujol A., Capdevila F., Moreno-García J., Moreno J., Mauricio J.C. (2020). Biological Processes Highlighted in *Saccharomyces cerevisiae* during the Sparkling Wines Elaboration. Microorganisms.

[13] Porras-Agüera J.A., Román-Camacho J.J., Moreno-García J., Mauricio J.C., Moreno J., García-Martínez T. (2020). Effect of endogenous CO_2_ overpressure on the yeast “stressome” during the “prise de mousse” of sparkling wine. Food Microbiol..

[14] Martínez-García R., García-Martínez T., Puig-Pujol A., Mauricio J.C., Moreno J. (2017). Changes in sparkling wine aroma during the second fermentation under CO_2_ pressure in sealed bottle. Food Chem..

[15] Bulletin officiel du Ministère de l’Agriculture et de l’Alimentation du 14 Novembre 2019. Cahier des Charges de l’Appellation d’Origine Contrôlée « Crémant de Loire » Homologué par l’Arrêté du 10 Octobre 2019, Publié au JORF du 19 Octobre 2019. https://info.agriculture.gouv.fr/gedei/site/bo-agri/document_administratif-baeb17d6-5e61-48e0-ab86-d4c28e387778.

[16] Bulletin Officiel du Ministère de l’Agriculture et de l’Alimentation du 3 Septembre 2020. Cahier des Charges de l’Appellation d’Origine Contrôlée «Champagne» Homologué par le Décret n°2010-1441 du 22 Novembre 2010, Modifié par Arrêté du 19 Août 2020 Publié au JORF du 27 Août 2020. https://info.agriculture.gouv.fr/gedei/site/bo-agri/document_administratif-f45cd2cf-c683-42c8-b1ea-c209314cfeb2.

[17] Valade M. (1999). La prise de mousse: Historique. Le Vigneron Champenois.

[18] Vizetelly H. (1882). A History of Champagne: With Notes on the Other Sparkling Wines of France.

[19] (2021). Union des Maisons de Champagne. https://maisons-champagne.com/en/appellation/stages-in-winemaking/from-still-wine-to-sparkling-wine/article/second-fermentation-capturing-the-sparkle.

[20] Esteruelas M., González-Royo E., Gil M., Kountoudakis N., Orte A., Cantos A., Fort F., Canals J.M., Zamora F. (2015). Influence of temperature during the second fermentation and aging of sparkling wine (Cava) on the properties of the foam. BIO Web Conf..

[21] Liger-Belair G., Bourget M., Villaume S., Jeandet P., Pron H., Polidori G. (2010). On the losses of dissolved CO_2_ during champagne serving. J. Agric. Food Chem..

[22] Liger-Belair G., Villaume S., Cilindre C., Jeandet P. (2009). Kinetics of CO_2_ fluxes outgassing from champagne glasses in tasting conditions: The role of temperature. J. Agric. Food Chem..

[23] Liger-Belair G., Parmentier M., Cilindre C. (2012). More on the losses of dissolved CO_2_ during champagne serving: Toward a multiparameter modeling. J. Agric. Food Chem..

[24] Liger-Belair G., Villaume S., Cilindre C., Polidori G., Jeandet P. (2009). CO_2_ volume fluxes outgassing from champagne glasses in tasting conditions: Flute versus coupe. J. Agric. Food Chem..

[25] Liger-Belair G., Conreux A., Villaume S., Cilindre C. (2013). Monitoring the losses of dissolved carbon dioxide from laser-etched champagne glasses. Food Res. Int..

[26] Liger-Belair G. (2005). The physics and chemistry behind the bubbling properties of Champagne and sparkling wines: A state-of-the-art review. J. Agric. Food Chem..

[27] Gonzalez Viejo C., Torrico D.D., Dunshea F.R., Fuentes S. (2019). Bubbles, foam formation, stability and consumer perception of carbonated drinks: A review of current, new and emerging technologies for rapid assessment and control. Foods.

[28] White M.R.H., Heymann H. (2015). Assessing the sensory profiles of sparkling wine over time. Am. J. Enol. Vitic..

[29] Torija M.J., Beltran G., Novo M., Poblet M., Guillamón J.M., Mas A., Rozes N. (2003). Effects of fermentation temperature and *Saccharomyces* species on the cell fatty acid composition and presence of volatile compounds in wine. Int. J. Food Microbiol..

[30] Martínez-García R., Moreno J., Bellincontro A., Centioni L., Puig-Pujol A., Peinado R.A., Mauricio J.C., García-Martínez T. (2021). Using an electronic nose and volatilome analysis to differentiate sparkling wines obtained under different conditions of temperature, ageing time and yeast formats. Food Chem..

[31] Caputi A.J., Ueda M., Walter P., Brown T. (1970). Titrimetric determination of carbon dioxide in wine. Am. J. Enol. Vitic..

[32] Moriaux A.L., Vallon R., Lecasse F., Chauvin N., Parvitte B., Zéninari V., Liger-Belair G., Cilindre C. (2021). How does gas-phase CO_2_ evolve in the headspace of champagne glasses?. J. Agric. Food Chem..

