# Supplementary material for: Does the Temperature of the prise de mousse Affect the Effervescence and the Foam of Sparkling Wines?
Source: Molecules. 2021 Jul 22;26(15):4434. doi: 10.3390/molecules26154434 (PMC8347939; doi:10.3390/molecules26154434)
Supplement: Supplementary file 1 [file molecules-26-04434-s001.zip › molecules-1272739-supplementary.pdf]

Article

Does the Temperature of the *prise de mousse* Affect the Effervescence and the Foam of Sparkling Wines?

Clara Cilindre [1,*], Céline Henrion [2], Laure Coquard [1,2], Barbara Poty [1], Jacques-Emmanuel Barbier [2], Bertrand Robillard [2] and Gérard Liger-Belair [1]

[1] Université de Reims Champagne-Ardenne, CNRS, GSMA UMR 7331, 51097 Reims, France; laure.coquard@gmail.com (L.C.); barbara.poty@univ-reims.fr (B.P.); gerard.liger-belair@univ-reims.fr (G.L.)
[2] Institut Œnologique de Champagne (IOC), ZI de Mardeuil, Epernay, 51530 Mardeuil, France; chenrion@iocwine.com (C.H.); jebarbier@iocwine.com (J.B.); brobillard@iocwine.com (B.R.)
* Correspondence: clara.cilindre@univ-reims.fr; Tel.: +33-326-91-32-58

Supplementary Materials

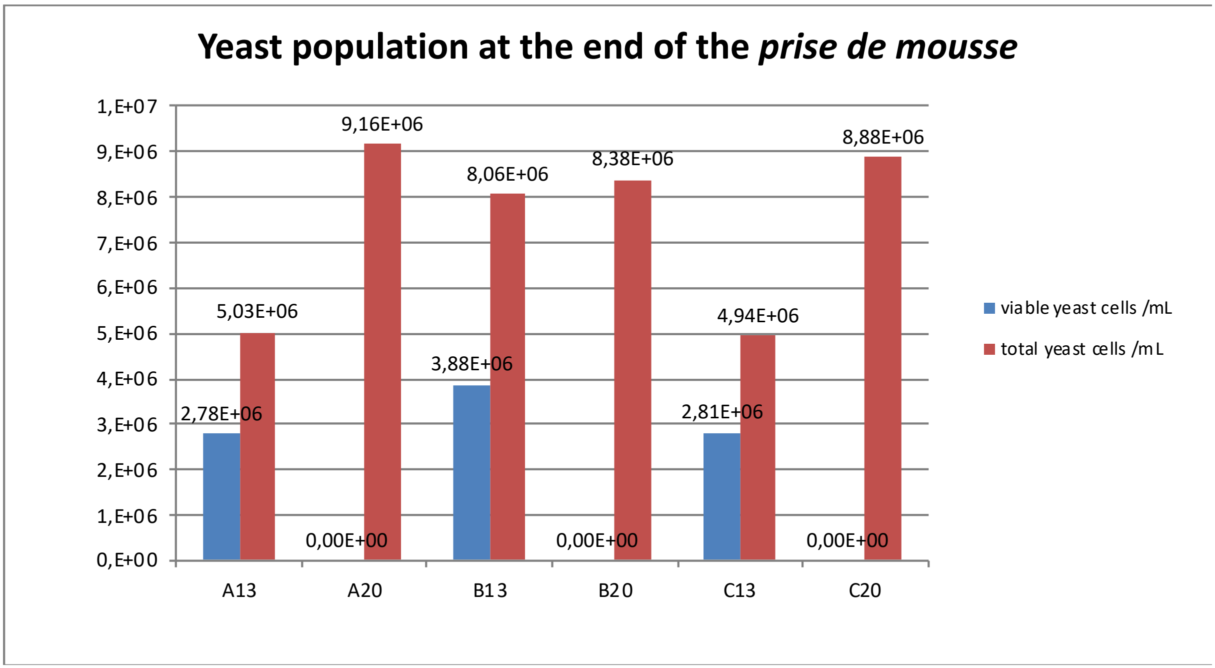

Figure S1. : Yeast population two weeks after the end of the *prise de mousse*. Yeast cells were counted according to the official OIV analytical method (OIV-MA-AS4-01).

**Enological parameter**	**A13**	**A20**	**B13**	**B20**	**C13**	**C20**
Alcohol (% v/v)	11.95	11.95	12.05	12.15	12.00	12.10
Reducing sugars (g/L)	1.90	1.50	1.70	1.00	2.20	1.20
Total acidity (g H_2SO_4 /L)	4.30	4.30	4.40	4.40	4.40	4.50
Total acidity (g tartaric acid /L)	6.60	6.60	6.70	6.70	6.70	6.90
pH	3.06	3.06	3.03	3.03	3.14	3.12
Volatile acidity (g H_2SO_4 /L)	0.32	0.30	0.29	0.28	0.30	0.29
Volatile acidity (g acetic acid /L)	0.39	0.37	0.36	0.34	0.37	0.36
Malic acid (g/L)	traces	traces	traces	traces	traces	traces
Total SO_2 (mg/L)	54.00	51.00	30.00	28.00	51.00	49.00
Free SO_2 (mg/L)	4.00	4.00	3.00	2.00	4.00	4.00
Turbidity (NTU)	0.52	0.55	1.28	0.54	1.67	0.69

Figure S2. : Enological parameters measured on sparkling wines two weeks after the end of the *prise de mousse* (n=1).
